# Our College Friends

**Published:** 1871-07

**Authors:** 


### OUR COLLEGE FRIENDS,

Say they will never again intrude their College trouble upon their readers. This is the very essence of wisdom unless they, in future, exercise more discretion in signing documents prepared by Council for their signatures.

They remind us very much of an old Georgia gentleman who was called upon to sign a document which he supposed involved only five dollars, to fix up a race-track. Having placed his signature to the document, he at once advanced the amount, supposing this to be the end of the transaction. On the following year he was called upon for his five dollars which he refused to pay. Having examined the document more closely, he found he was "sucked in" for five dollars for ten years, which he was compelled to pay at the end of the law.

In the couse of a few years, at a sweeping revival, the old gentleman embraced religion, and the minister having called upon him for his name to be enrolled upon the church-book, he replied, "Brother S., is there any writing about it?" "No," said the minister, "I merely want your name enrolled on the church-book." "Well," said the old man, "my brother, I feel like my hope is well-grounded; I believe I have undergone a change; that I am on the Lord's side—but, sir, do you say there's any writing about it?" To which the minister replied,—"Oh! nothing, but to simply put your name on the church-book." "Ah, but my brother, I can't do that—I signed a document once that brought my gray hairs almost in sorrow to the grave—I can't sign any more papers. I am sorry it is so. I would like to associate with God's people, but if there is any writing about it, I must back out—I can't sign any more papers. Bless you, I've seen too much trouble; I can't sign any more papers, brother S."

So with our friends of the *Atlanta Medical and Surgical Journal.* They have signed and assumed the responsibility of so many documents containing misstatements that, like brother B., they have concluded that, notwithstanding their hope is well grounded, "if there is any writing about it," they would wish to be considered "out of it." May the good Lord bless them in their "new departure," and the profession throw the veil of charity over their past "signing."

